# Fatal injury as a function of rurality-a tale of two Norwegian counties

**DOI:** 10.1186/1757-7241-21-14

**Published:** 2013-03-02

**Authors:** Håkon Kvåle Bakke, Ingrid Schrøder Hansen, Anette Bakkane Bendixen, Inge Morild, Peer K Lilleng, Torben Wisborg

**Affiliations:** 1 Anaesthesia and Critical Care Research Group, Faculty of Health Sciences, IKM, University of Tromsø, Tromsø 9037, Norway; 2 Section of Pathology, The Gade Institute, University of Bergen, Bergen, Norway; 3 Department of Pathology Haukeland University Hospital, Bergen, Norway; 4 Hammerfest Hospital, Department of Anaesthesiology and Intensive Care, Finnmark Health Trust, Hammerfest, Norway

**Keywords:** Trauma, Epidemiology, Rural, Urban, Road traffic injuries, Injury

## Abstract

**Background:**

Many studies indicate rural location as a separate risk for dying from injuries. For decades, Finnmark, the northernmost and most rural county in Norway, has topped the injury mortality statistics in Norway. The present study is an exploration of the impact of rurality, using a point-by-point comparison to another Norwegian county.

**Methods:**

We identified all fatalities following injury occurring in Finnmark between 2000 and 2004, and in Hordaland, a mixed rural/urban county in western Norway between 2003 and 2004 using data from the Norwegian Cause of Death Registry. Intoxications and low-energy trauma in patients aged over 64 years were excluded. To assess the effect of a rural locale, Hordaland was divided into a rural and an urban group for comparison. In addition, data from Statistics Norway were analysed.

**Results:**

Finnmark reported 207 deaths and Hordaland 217 deaths. Finnmark had an injury death rate of 33.1 per 100,000 inhabitants. Urban Hordaland had 18.8 deaths per 100,000 and rural Hordaland 23.7 deaths per 100,000. In Finnmark, more victims were male and were younger than in the other areas. Finnmark and rural Hordaland both had more fatal traffic accidents than urban Hordaland, but fewer non-fatal traffic accidents.

**Conclusions:**

This study illustrates the disadvantages of the most rural trauma victims and suggests an urban-rural continuum. Rural victims seem to be younger, die mainly at the site of injury, and from road traffic accident injuries. In addition to injury prevention, the extent and possible impact of lay people’s first aid response should be explored.

## Introduction

Injury is a leading cause of death worldwide, accounting for 16% of the global burden of disease [1]. Commonly affecting otherwise healthy individuals, injury is the most frequent cause of death among people under the age of 40 years in Norway [2].

It is well established that rural areas have higher injury-related mortality rates than urban areas [3-5], with higher death rates from drowning, fire, and especially traffic accidents [3,4]. High-risk occupations (i.e. farming, mining, and fishing), greater alcohol con`sumption, attitudes towards risk reducing measures, and lower socio-economic status have been discussed [3,6,7] as risk factors. A larger proportion of rural trauma victims die at the scene of injury, which is credited to longer discovery, response, and transport times [8,9].

Finnmark lies at the very north end of the Scandinavian Peninsula, in Norway. Covering an area roughly the size of Denmark, today it is home to a mere 73,694 people [10]. For decades, this sparsely populated region has had death rates from injury well above the national average.

We have previously described injuries in Finnmark and investigated their changes over time, and Finnmark seemed to follow a typical rural injury pattern [11,12]. However, as there are several other counties in Norway that are also rural or partly rural, this alone should not account for Finnmark’s high death rate [13].

The aim of this study was to explore the impact of different degrees of rurality on the epidemiology of trauma. This was achieved through a point-by-point comparison of Finnmark to another Norwegian county based on a thorough analysis of a two-year patient material. To extend the material and reduce the risk of random variations, we also analysed registry data for a ten-year period from Statistics Norway.

## Methods

### Inclusion and exclusion

All deaths from external causes (ICD-10 V01–Y98) occurring in Finnmark County during the five-year period from 1 January 2000 through 31 December 2004, and occurring in Hordaland County in the two-year period from 1 January 2003 through 31 December 2004 were obtained from the Norwegian Cause of Death Registry. The Cause of Death Registry routinely records all deaths in Norway and codes the deaths in accordance to the ICD-10 system based on the information from the death certificate and autopsy reports, thus ensuring that the coding is performed uniformly. We also included those injury cases that occurred in Finnmark or Hordaland where the patient succumbed after being transferred to a hospital outside of the county.

We excluded deaths from isolated, simple fractures after a fall at ground level that occurred in persons aged over 64 years, and those from poisonings. Victims of simultaneous trauma and intoxication were included. The criteria used were the same as for our previous studies of Finnmark, and were originally chosen to ensure comparability to similar studies [11,12].

### Data collection and definitions

Information concerning cause of injury, time and place of death, and demographic data was obtained from ambulance and hospital records and/or police and autopsy reports (where available), and were recorded in a standard form.

Death was defined as the point in time where the patient became lifeless and no attempt at resuscitation was made or such attempts were terminated. As such, death did not require a physician to declare the patient dead. Time from injury to death was the time that elapsed from the injury up to this point. Place of death was set as the place this occurred as stated in the patient’s records.

Health statistics regarding the major causes of death were obtained from Statistics Norway for the period from 1 January 2001 through 31 December 2009. These data were not collected specifically for the study, but the decision to access them was made prior to the collection of the primary data. In figures and tables where Statistics Norway is the source, this is stated clearly in the legends.

### The study areas

To investigate rural/urban differences, we divided our material from Hordaland County into two categories; injuries that occurred inside the municipality of Bergen (urban), and injuries that occurred outside of Bergen (rural).

At the beginning of the study period, the urban Hordaland subgroup had 235,423 inhabitants (density 506/km^2^), which were all served by the University Hospital of Bergen. The rural Hordaland subgroup had 206,237 inhabitants (density 14/km^2^), which were served by three local hospitals; Odda, Voss, and Stord, as well as the University Hospital of Bergen. Finnmark had 76,629 inhabitants at the beginning of the study period and 73,210 at its end (density 1.5/km^2^). Finnmark is served by two local hospitals, Hammerfest, and Kirkenes, as well as the University Hospital of Tromsø, which is located outside the county. Except for the differences in area and population density, the trauma systems in Finnmark and Hordaland are otherwise rather similar; there are similar requirements for level of education in the EMS and response times. Both counties have helicopter service available as part of EMS.

### Statistical analysis

SPSS version 16.0 was used for statistical analysis. The Mann-Whitney U test, Kruskal-Wallis test, or analysis of variance (ANOVA) was used for continuous data. For categorical data and rates, a chi-square test or Fisher’s exact test was used. Comparison of groups was two-tailed with statistical significance chosen at p < 0.05.

### Ethics

Approval for the study was given by the Norwegian Directorate for Health and Social Affairs (07/4817), the Norwegian Data Inspectorate (07/01595-3/clu), the Privacy Ombudsman for Research (17430/2/LT), the Norwegian Director of Public Prosecutions (Ra 07-526 IFO/mw 639.2), and the Regional Committee for Medical and Health Research Ethics (200702984-3/IAY/400).

## Results

### General characteristics

**Figure 1:**
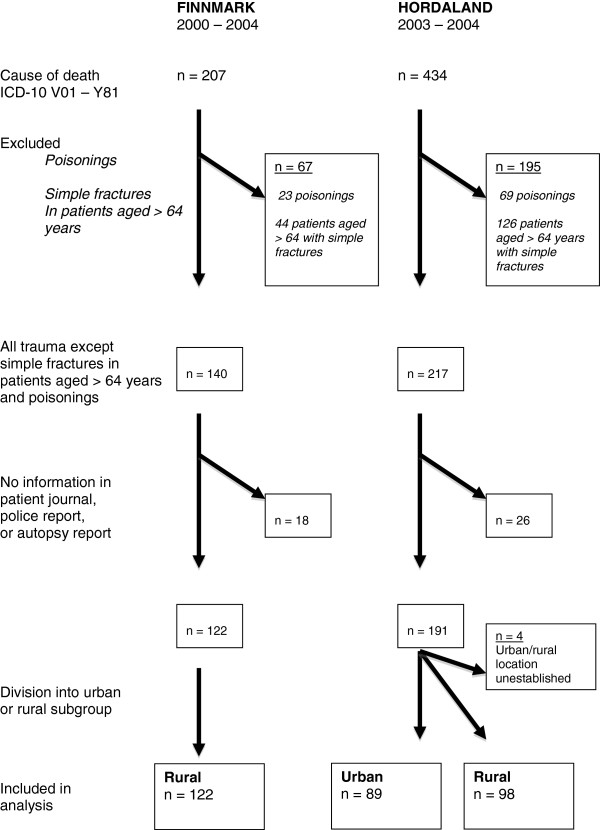
Flow diagram illustrating the inclusion and exclusion process.

The process of inclusion and exclusion is presented in Figure 1.

The division of Hordaland into rural and urban groups was done according to the information found in the patients’ journals. Therefore, we could not calculate the mortality rate for the groups based on the cases included, but had to use the mortality rate based upon the cases found. We found 89 deaths in urban Hordaland and 98 deaths in rural Hordaland during the study period. Finnmark had an autopsy rate of 34% (n = 48) and Hordaland had a rate of 80% (n = 173). The mortality rates, age, and gender distribution are given in Table 1.

**Table 1:** Main characteristics of the patients by study area

	**Finnmark**	**Rural Hordaland**	**Urban Hordaland**
Total death rate	33.1/100,000^a,b^	23.7/100,000	18.8/100,000
Median age (inter quartile range)	40 (27–55)^a,b^	50.5 (33–71)	46 (32–66)
Male gender	80%	75%	76%
Pre-hospital death	85%^b^	82%	72%

^a^ Statistically significant difference from rural Hordaland.

^b^ Statistically significant difference from urban Hordaland.

The mortality rate was significantly higher in Finnmark compared with the two other areas (p < 0.001 against urban and p = 0.013 against rural Hordaland). No significant difference in rates was observed between urban and rural Hordaland (p = 0.124). The fatality victims in Finnmark were younger aged than those in the other areas (p = 0.022 against urban and p = 0.006 against rural Hordaland), but there was no difference in age within Hordaland (p = 0.072). There were no statistically significant differences in gender distribution (p = 0.493) between the groups.

### Time and place of death

**Figure 2:**
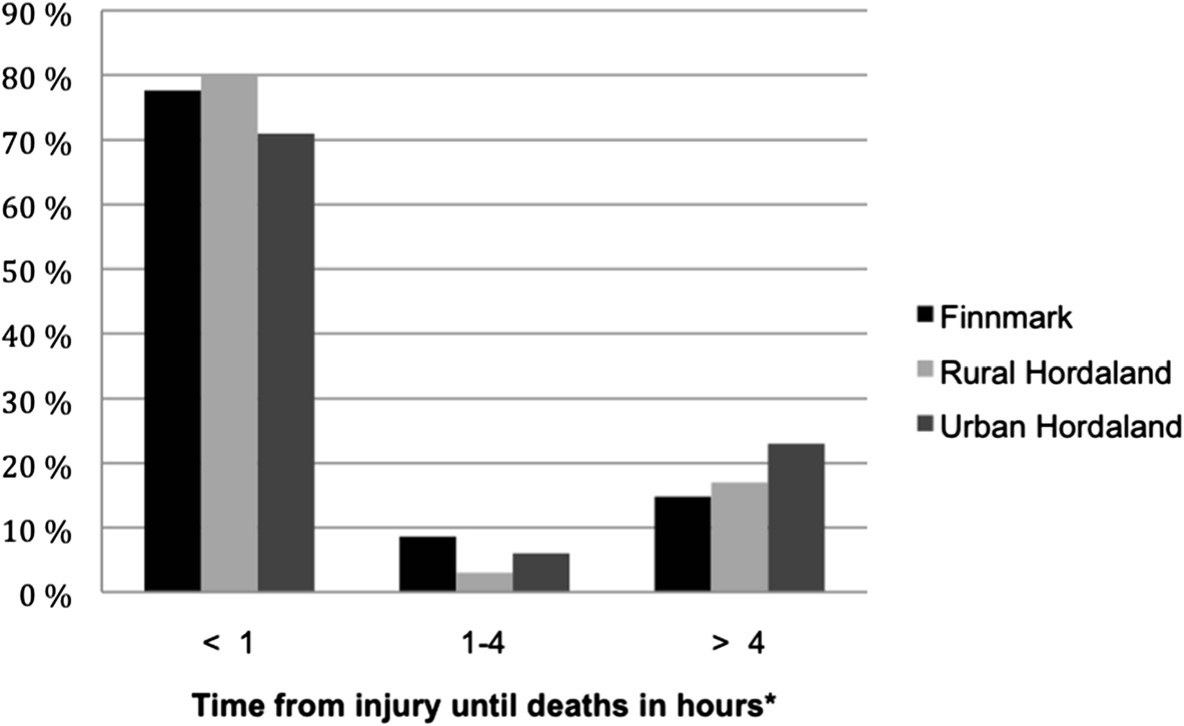
Comparison of the distribution of the time from injury until death among the three study areas (hours). * Based on 81/122 patients in Finnmark, 86/89 in urban and 95/98 in rural Hordaland. For the excluded patients, time could not be determined with certainty although all died at the scene of injury and likely within one hour after injury.

The time from injury until death could be established for 81 (66%) cases in Finnmark and 86 (97%) and 95 (97%) cases in urban and rural Hordaland, respectively (Figure 2). There was no difference in time distribution between the areas. However, all of the fatalities where time from injury to death could not be established occurred at the scene of injury, likely within the first hour of injury.

The place of death is shown in Table 2. When comparing urban Hordaland to Finnmark, there were fewer pre-hospital deaths in urban Hordaland (72% vs. 85%, p = 0.018); there was no difference between rural Hordaland and Finnmark or within Hordaland in this regard.

**Table 2:** Comparison of place of death between the study areas

	**Finnmark**	**Rural Hordaland**	**Urban Hordaland**
*Pre-hospital (total)*	*104 (85%)*	*80 (82%)*	*64 (72%)*
At injury site	101 (83%)	78 (80%)	64 (72%)
During transport	3 (2%)	2 (2%)	0 (0%)
*In-hospital (total)*	*16 (13%)*	*16 (16%)*	*25 (28%)*
Emergency room	6 (5%)	2 (2%)	3 (3%)
During admission	10 (8%)	14 (14%)	22 (25%)
*After discharge*	1 (1%)	2 (2%)	0 (0%)
*Not established*	1 (1%)	0 (0%)	0 (0%)

### Cause of injury

**Figure 3:**
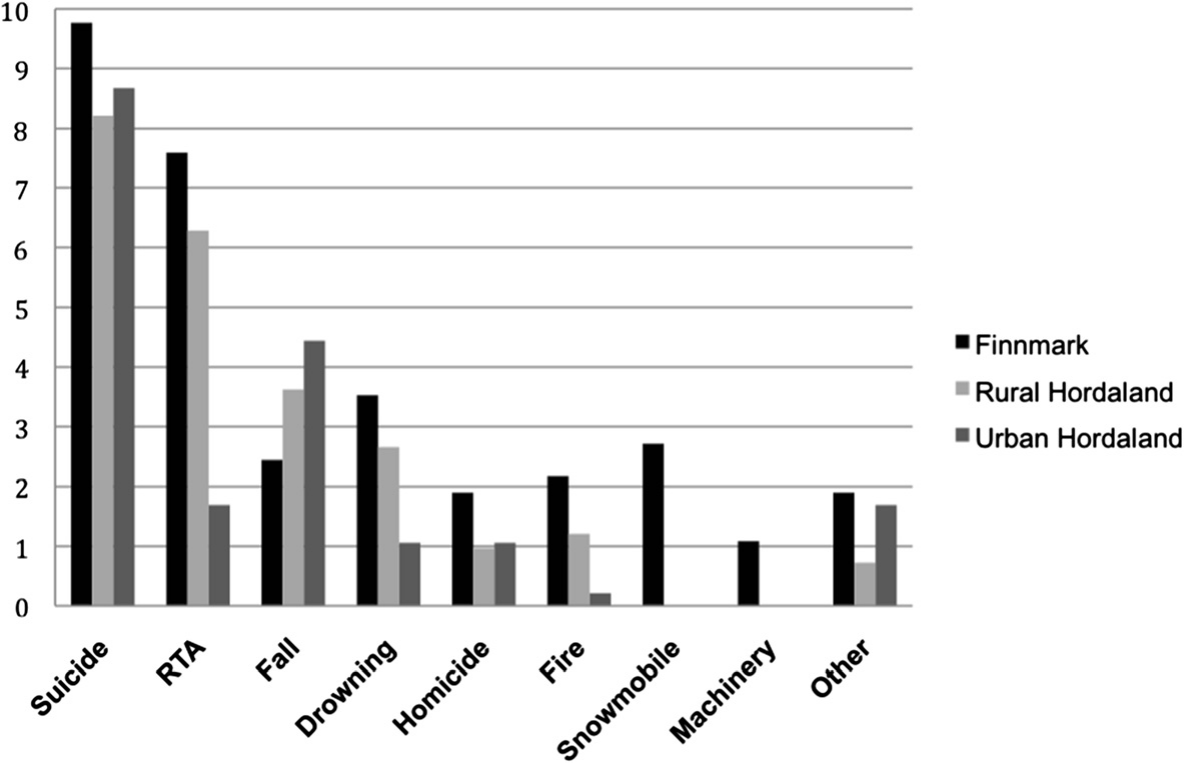
**Distribution of the cause of injury in the study areas (rates are deaths per 100,000 inhabitants per year). **RTA = road traffic accidents.

The distribution of the cause of injury within each area is given in Figure 3, and the cause of injury specific death rates are given in Table 3. Finnmark had more deaths from road traffic accidents (p < 0.001), snowmobile accidents (p < 0.001), fires (p = 0.007), drowning (p = 0.015), and machinery (p = 0.037) than urban Hordaland. Rural Hordaland had more deaths from road traffic accidents than urban Hordaland (p = 0.008) with a rate of 6.3 and 1.70 per 100,000 inhabitants, respectively.

**Table 3:** Cause of injury-deaths per 100,000 inhabitants by area

	**Finnmark**	**Rural Hordaland**	**Urban Hordaland**
Cause of injury			
*Suicide*	*9.8*	*8.2*	*8.7*
*RTA*	*7.6*	*6.3*	*1.7*
*Fall*	*2.4*	*3.6*	*4.4*
*Drowning*	*3.5*	*2.7*	*1.1*
*Homicide*	*1.9*	*1.0*	*1.1*
*Fire*	*2.2*	*1.2*	*0.2*
*Snowmobile*	*2.7*	*0*	*0*
*Machinery*	*1.1*	*0*	*0*
*Other*	*1.9*	*0.7*	*1.9*

There was no difference between rural Hordaland and Finnmark except for deaths from snowmobile accidents (p = 0.001) and machinery (p = 0.049); rural Hordaland had none.

In rural Hordaland, 7 of 98 accidents occurred at work, and there was no difference (p = 0.415) in these numbers compared to Finnmark (11 of 122) when adjusted for employment rate. Compared to urban Hordaland (0 of 89), both of the rural areas had a higher share of accidents occurring at work (p = 0.01 and p < 0.001, in rural Hordaland and Finnmark respectively).

### Data from Statistics Norway

**Figure 4:**
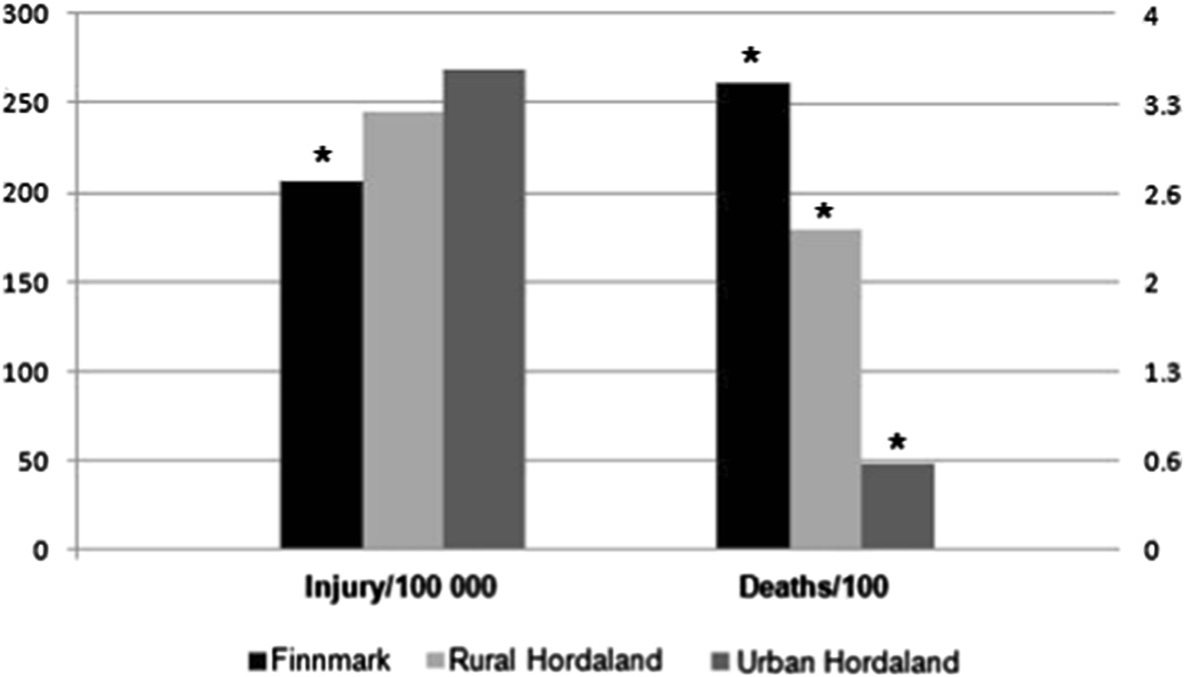
Road traffic accident data from Statistics Norway for the period 2001 to 2009 involving personal injury: accident rate per 100,000 inhabitants, and deaths per 100 accidents with personal injury per year for each study area. * Statistically significant difference between study areas (p < 0.05).

From 2001 through 2009, Statistics Norway provided the number of suicides, falls, and road traffic accidents with personal injury. Finnmark had a higher suicide rate (p < 0.01) than the other areas at 14.6 deaths per 100,000 inhabitants per year compared with 10.5 deaths per 100,000 inhabitants per year in urban and 8.9 deaths per 100,000 inhabitants per year in rural Hordaland (no difference, p = 0.43). Road traffic accidents are displayed in Figure 4. When adjusted for population, Finnmark had an annual rate of 206 road traffic accidents with injury per 100,000 inhabitants per year. Mortality, defined as number of deaths per 100 road traffic accidents (RTA) with personal injury, was 3.5 per 100 in Finnmark. Rural Hordaland had 245 accidents per 100,000 inhabitants, and 2.4 persons killed per 100 accidents. Urban Hordaland had a rate of 269 RTA with injury per 100,000 inhabitants per year, and 0.64 persons killed per 100 accidents. The rate of accidents was lower for Finnmark compared with both rural and urban Hordaland (p = 0.003 and p = 0.001, respectively), whereas it did not vary significantly among urban or rural Hordaland (p = 0.109). The number of persons killed per 100 accidents was higher for Finnmark than rural Hordaland (p = 0.029), which in turn was higher than urban Hordaland (p < 0.001). For fall injuries, Finnmark had a rate of 9.9, rural Hordaland 10.9, and urban Hordaland 12.6 deaths per 100,000 inhabitants per year. These were not significantly different, but there was a slight trend, with urban Hordaland comparing to Finnmark at p = 0.08 and to rural Hordaland at p = 0.13.

## Discussion

This study shows that inhabitants of the most rural and remote study area in Norway, Finnmark County, have a significantly increased risk of dying from injuries compared with Norwegians in less rural and urban areas. The findings indicate a gradient from urban to rural areas where rurality in itself increases risk of death when injuries occur. Rural victims tend to be younger and succumb at the scene of the accident. A major finding is the fact that the prevalence of non-fatal RTAs seem to be lower in the most rural area, indicating that the risk of dying from an RTA is considerably increased.

Suicide was the greatest cause of death in all areas. There was a significant difference between Finnmark and the other areas in the data from Statistics Norway. This category did not follow an urban-rural gradient, which is in line with higher suicide rates being reported in rural communities [3,14,15], whereas other studies have identified this as an urban problem [16]. This study does not report suicides committed by poisoning, and thus the complete picture is not provided. One might speculate in a rural-urban difference in suicide methods with rural inhabitants employing more traumatic methods. Anyway, the suicide rate may help explain why Finnmark has a higher trauma death rate than other rural areas.

Road traffic accidents comprised the second largest cause of death. The data we obtained from Statistics Norway indicated that there are more injuries in urban Hordaland, but more deaths in rural Hordaland and Finnmark. The discrepancy between fatality and injury rates may be due to differences in accident severity, disadvantages in rural trauma care, or both. Although urban areas may have higher injury rates due to higher traffic density, they may also have lower accident severity as a result of lower speeds. Conversely, rural accidents may be more severe owing to higher speeds, but possibly also to more head-on collision, older, less crash-secure cars; and different attitudes towards seatbelt use [3,17]. Disadvantages in rural trauma care may come from longer discovery and transport times due to long distances or weather conditions [8], or health care workers in rural areas may be less experienced in handling trauma because they see fewer cases than their urban colleagues [18]. Reference to a non-trauma centre has been shown to adversely affect outcome in trauma [19]. In an area as rural as Finnmark, direct transfer is often not an option, which in turn may have an impact on mortality rate. Local hospitals in Finnmark have improved in-house trauma care by the regular multiprofessional trauma team training that seems to even out differences between urban and rural care [20]. Still, studies from Northern Norway have shown areas of potential improvement in local hospital trauma management concerning transfer routines, damage-control surgery, and use of diagnostic imaging [21,22]. These conditions are not exclusive to Finnmark, indicating that distance in itself probably plays a part in the disadvantages of the most rural trauma victims. This is supported by recent findings in Norway concerning paediatric trauma-related death [13]. Besides a higher death rate from RTA, both rural areas in our study exceeded the urban area for rates of fatal injuries occurring at work. In addition, Finnmark has a higher rate of death from fires and drowning than urban Hordaland, and a higher share of pre-hospital deaths. For these two variables, rural Hordaland did not significantly differ from the two other areas, possibly putting it somewhere between them. In the material from Statistics Norway on RTAs, rural Hordaland also fell between the other two areas, and for falls (the third largest cause of injury) there was a similar though non-significant trend. These variables constitute a typical rural picture. Therefore, this study displays an urban-rural continuum, with rural Hordaland more rural than Urban Hordaland, and Finnmark more rural than rural Hordaland, which indicates that part of Finnmark’s problem is its highly rural nature.

The inclusive trauma system, encompassing prevention, legislation, and health care, has been shown to reduce mortality [23,24]. The Scandinavian trauma system has been regarded as immature, and implementation of a system customised to the local conditions has been called for [25]. This study highlights some of the challenges the trauma system must be designed to face to meet the needs of rural Scandinavia. Given the large share of those dying at the scene, the focus of the system must above all be on prevention. Also, little is known about the extent and possible influence of first responder activities at the site of injury and trauma [26]. It is possible that an increased focus on layperson first response at the site of injury might mitigate the effect of long distances between the injury site and professional help. First responder training has been effective in low-income countries [27,28]. The same effects of distance upon response times may hamper first responder groups just as they have the EMS, and raising the level of first-aid knowledge in the general populace may be worth investigating.

Finnmark differed from both urban and rural Hordaland in deaths from machinery and snowmobile accidents. In Norway, snowmobiles are least used in the western part, and most commonly used in the northern counties. Snowmobile accident is the fourth largest cause of death in Finnmark and could explain some of the differences in total death rates among the counties.

Further research should try to establish the survivability of the rural injuries. Such an analysis was not performed in this study owing to the low autopsy rate in Finnmark. The local police determine what cases are sent for autopsy and the selection is therefore unlikely to be representative. Likewise, cause of death and preventability would be of interest in future studies.

This study has several limitations. First, the observation period was short, especially in Hordaland with only two years. With a short observation period, there is the risk of hitting peaks or troughs of fluctuating trends, thereby obtaining misleading results. Dividing Hordaland into rural and urban subgroups was necessary to explore the issue of rural character, but it also meant reducing the number in each group when comparing subgroups such as place or mode of accident. The difference in autopsy rates, 80% in Hordaland and a mere 34% in Finnmark, is also a concern, although the endpoints of this study are rather robust despite the lack of autopsy.

## Conclusion

This study illustrates the disadvantages of the most rural trauma victims, and suggests an urban-rural continuum. In addition to injury prevention and the implementation of a customised trauma system, the extent and possible impact of lay people’s first response should be explored.

## Consent

The Regional Committee for Medical and Health Research Ethics of Northern Norway approved the study (ref. 200702984-3/IAY/400 and 2010/1703-4) and exempted the authors from obtaining consent from the next-of-kin of the victims of traumatic death studied in this paper.

## Competing interests

None of the authors have any conflicts of interest to declare.

## Authors’ contributions

HKB participated in developing the design, carried out the data collection for Finnmark, performed the statistical analysis, and drafted the manuscript. ISH carried out the data collection for Hordaland, and revised the manuscript. ABB carried out the data registration for Hordaland, and revised the manuscript. IM facilitated and supervised the data registration for Hordaland, and revised the manuscript. PKL facilitated and supervised the data registration for Hordaland, and revised the manuscript. TW conceived of the study, developed the design, supervised the registration of data in Finnmark and analysis and interpretation of data, and helped draft the manuscript. All authors read and approved the final manuscript.
